# Uricase Crowding via Polyelectrolyte Layers Coacervation for Carbon Fiber-Based Electrochemical Detection of Uric Acid

**DOI:** 10.3390/polym14235145

**Published:** 2022-11-26

**Authors:** Anna A. Baldina, Liubov V. Pershina, Ulyana V. Noskova, Anna A. Nikitina, Anton A. Muravev, Ekaterina V. Skorb, Konstantin G. Nikolaev

**Affiliations:** Infochemistry Scientific Center, ITMO University, 191002 Saint Petersburg, Russia

**Keywords:** layer-by-layer assembly, polyelectrolyte, crowding effect, uric acid, SARS-CoV-2

## Abstract

Urate oxidase (UOx) surrounded by synthetic macromolecules, such as polyethyleneimine (PEI), poly(allylamine hydrochloride) (PAH), and poly(sodium 4-styrenesulfonate) (PSS) is a convenient model of redox-active biomacromolecules in a crowded environment and could display high enzymatic activity towards uric acid, an important marker of COVID-19 patients. In this work, the carbon fiber electrode was modified with Prussian blue (PB) redox mediator, UOx layer, and a layer-by-layer assembled polyelectrolyte film, which forms a complex coacervate consisting of a weakly charged polyelectrolyte (PEI or PAH) and a highly charged one (PSS). The film deposition process was controlled by cyclic voltammetry and scanning electron microscopy coupled with energy-dispersive X-ray analysis (at the stage of PB deposition) and through quartz crystal microbalance technique (at latter stages) revealed uniform distribution of the polyelectrolyte layers. Variation of the polyelectrolyte film composition derived the following statements. (1) There is a linear correlation between electrochemical signal and concentration of uric acid in the range of 10^−4^–10^−6^ M. (2) An increase in the number of polyelectrolyte layers provides more reproducible values for uric acid concentration in real urine samples of SARS-CoV-2 patients measured by electrochemical enzyme assay, which are comparable to those of spectrophotometric assay. (3) The PAH/UOx/PSS/(PAH/PSS)_2_-coated carbon fiber electrode displays the highest sensitivity towards uric acid. (4) There is a high enzyme activity of UOx immobilized into the hydrogel nanolayer (values of the Michaelis–Menten constant are up to 2 μM) and, consequently, high affinity to uric acid.

## 1. Introduction

Macromolecular crowding plays a key role in the cellular environment affecting gene expression [1], protein stability [2], and the kinetics of enzyme reactions [3,4,5], to name just a few. This is due to dynamic compartmentalization via liquid–liquid phase separation, which increases the local enzyme concentration and alters the diffusion of substrates. From thermodynamic viewpoint, in agreement with the McMillan and Mayer theory of particle–particle interaction, entropy decreases when proteins and other macromolecules are excluded from the physical volume occupied by bulky crowding agents.

Enzymatic compartments could be developed through encapsulation in polymer nanoparticles [6] or surface treatment methodologies, such as immobilization onto Langmuir–Blodgett [7] or Langmuir–Schaefer films [8]; the latter, however, require expensive equipment and highly qualified personnel. Alternatively, layer-by-layer (LbL) assembled nanoarchitectures are easily accessible through adsorption of polyelectrolytes on the electrode [9,10,11,12,13], which could avoid a decrease in the mobility of enzyme binding sites [14,15,16,17]. Pairs of synthetic polyelectrolytes allow one to develop such crowded reproducible conditions due to the formation of the individual phase of the coacervate complex and exclusion of excess salt and water from the phase [18,19]. Therefore, the kinetics of enzymatic reactions in crowded medium does not depend on the ionic strength due to free ions, it rather depends on the type of polyelectrolyte macromolecules that surround the enzyme and their composition [20,21]. Theoretical predictions of the decrease of the effective Michaelis constant with an increase in the volume fraction of crowding agents [4] agree with the experimental findings that the highest activity of horseradish peroxidase and urate oxidase is achieved in aqueous two-phase system of high molecular weight dextran and low molecular weight poly (ethylene glycol), and partition of these enzymes between phases is a function of salt concentration and ion position in Hofmeister series [5].

We have previously suggested an approach for electrochemical immunosensors with a quasi-mobile polyelectrolyte–protein hydrogel interface [22], including nanofilms [23]. Hydrogel interfaces based on neutral polyelectrolytes provide reproducible results in complex systems and simple variation of hydrogel composition, which enables facile data collection for machine learning to decrease the limit of detection [22,24]. The crowded tick-borne encephalitis antibodies immobilized in the electrode of polyelectrolyte-protein design could detect up to five whole virus particles. It proves polyelectrolyte effectiveness for the sensitivity increase of the immunosensors compared to the antibody-only modified electrode. Hydrophilic properties of polyelectrolytes in a crowded medium suppressed nonspecific protein adsorption [23]. We have shown that LbL assembly effectively controls the adsorption of antigens and antibodies conjugated with horseradish peroxidase on the electrode surface. Even though theoretical works on enzyme behavior in crowded environments indicate unique properties of hydrogels made from charged macromolecules (e.g., a topologically frustrated state of DNA in poly (styrene sulfonate) (PSS) hydrogel [25]), mainly uncharged species were experimentally studied [26]. Moreover, the entropy of such crowded systems is predicted to be higher than that in aqueous solutions [25,27].

In this work, we propose a polyelectrolyte crowding concept for the design of an enzyme-based sensor towards uric acid (UA), which was recently identified as a prognostic indicator and a marker of disease severity in COVID-19 patients [28]. We show that UOx, noncovalently immobilized into hydrogel nanolayers, preserves its reactivity and enzyme-based electrochemical biosensor, with the ability to detect micromolar concentrations of uric acid, could be developed.

## 2. Materials and Methods

Carbon fibers (CFs) with surface density of 450 g/m^2^ were produced by M-Carbo (Minsk, Belarus). Lyophilized *Bacillus fastidiosus* uricase (9 U/mg), UA, branched polyethyleneimine (PEI, M_W_~750,000, 50% (*w*/*v*) solution), PSS (M_W_~1,000,000), poly (allylamine hydrochloride) (PAH, M_W_~15,000–20,000), 2-nitrophenyl octyl ether (*o*-NPOE), high molecular weight poly (vinyl chloride) (PVC), and 45 wt% THF solution in H_2_O (Sigma-Aldrich, Burlington, USA) and HCl and KCl (Fluka, Buchs, Switzerland) were used as received. Silver conductive adhesive (Kontaktol, Keller, Saint Petersburg, Russia) and nail polish were purchased from local stores. All solutions were prepared using deionized (DI) water (>18.0 MΩ cm, Millipore Milli Q Gradient System, Burlington, USA). Urine samples were provided by the Children’s City Clinical Hospital No. 5, named after N.F. Filatov (Saint Petersburg, Russia), and a 10-fold dilution with a buffer solution (pH 7.4) was applied to the samples before measurements.

Carbon fiber electrodes (CFEs) were modified with Prussian blue Fe_4_[Fe(CN)_6_]_3_ (PB) through electrodeposition using 12 cyclic scans of cyclic voltammetry within the range of –0.2 to 0.6 V at a scan rate of 0.05 V/s from a solution containing 1.5 mM K_3_[Fe(CN)_6_] and 1.5 mM FeCl_3_ in 0.1 M KCl and 0.1 M HCl. Then, the CF/PB coating was washed with DI water and activated by applying another 50 cycles in electrolyte solution (0.1 M KCl and 0.1 M HCl) using the same protocol. After deposition, the electrodes were washed with DI water and dried in air. Surface morphology and elemental composition of PB-coated CFEs were studied on a Tescan Vega-3 scanning electron microscope (Brno, Czech Republic) equipped with an energy-dispersive X-ray analyzer (EDX).

The films of (PEI/UOx)(PEI/PSS)_n_ (n = 1, 2) and (PAH/UOx)(PAH/PSS)_2_ were deposited onto the working surface of the PB-coated CF electrode using the LbL technique, with an immersion of the electrode into the solution of corresponding component for 30 s and washing with PBS (pH 7.4) before depositing the subsequent layer. This pH value was chosen due to the maximum catalytic activity (optimum pH is 7) and stability of uricase under these conditions and that it falls within pH range of real urine samples (pH 4.5–8.0).

Cyclic voltammograms were recorded using an SP-50 potentiostat-galvanostat (Electrochemical Instruments, Chernogolovka, Russia) in a standard three-electrode cell at 25 °C. CF/PB/(PEs/UOx)_n_ (n = 1, 2) was used as the working electrode; CF/Ag/AgCl/PEs, the reference; and CF, a counter electrode.

QCM measurements were run using a flow module connected to a 10 M eQCM instrument (Gamry Instruments, Warminster, PA, USA). The electrodes were soaked in piranha solution for 1 min, washed with DI water, and dried under inert gas before use. The 5-MHz Au-coated AT-cut quartz crystal (Shenzhen RenLux Crystal Company, Shenzhen, China) was fixed using crystal holder. All solutions were flowed into the modules using a BT100LC peristaltic pump (Baoding Chuangrui Precision Pump Company, Baoding, China) at a flow rate of 2 mL/min. To correlate the change of oscillation frequency of quartz microcrystal, ∆*f*, and the mass of deposited PE or UOx layers, ∆*m*, Sauerbrey Equation (1) was used:

(1) $$∆f=\frac{2f_{0}^{2}}{A\sqrt{\rho_{q}\mu_{q}}}∆m,$$

where *f*_0_ is the fundamental frequency (5 MHz); *A* is the piezoelectrically active crystal area (1.5386 cm^2^); *ρ*_q_ is the density of quartz (2.648 g·cm^−3^); and *μ*_q_ is the shear modulus of quartz (2.947 × 10^11^ g·cm^−1^·s^−2^).

The Ag/AgCl reference electrode was prepared by covering with polyelectrolytes and Ag-based ink. CF was covered with nail polish leaving 15 mm uncovered at both ends. Following this step, one of the ends was coated with Ag conductive glue and left to dry in air for 30 min. Then, a Ag-coated area was immersed into 1 M HCl and rinsed with DI water to produce a AgCl-coated CF. The working area of the electrode was modified via LbL assembly as follows. Firstly, the electrode was dipped for 30 s into PEI solution and, then, for 30 s into PSS solution, and it was rinsed with DI water between dips. Thus, a total of 32 layers of PEI and 32 layers of PSS were adsorbed. The membrane precursor was prepared by stirring the solution of 335 mg of *o*-NPOE and 165 mg of PVC in 1 mL of THF for 20 min and then stored at 4 ± 2 °C before use. The polymer-coated end of the fiber was dipped into the membrane mixture four times with 2-min intervals for drying. Finally, the electrode was suspended vertically for membrane setting for 40–90 min at 25 °C.

To evaluate the kinetics of the enzymatic process, the Michaelis–Menten constant was calculated for each type of structural modification according to Equation (2) [29]:

(2) $$\frac{1}{I_{\mathrm{ss}}}=\frac{K_{m}}{I_{\max}}\frac{1}{C}+\frac{1}{I_{\max}},$$

where *K*_m_ is the Michaelis–Menten constant, µM; *I*_max_ is the limiting current, µA; *C* is the substrate concentration, µM; and *I*_ss_ is the current of the reaction, µA.

Spectrophotometric determination of UA was conducted using a Klinitest-MK reagent set (Eko-servis, Saint Petersburg, Russia). This set exploits the reaction of 4-aminoantipyrine, 2-hydroxy-3,5-dichloro-benzenesulfonate, and H_2_O_2_ in the presence of peroxidase that affords a colored product (N-(4-antipyryl)-3-chloro-5-sulfonate-*p*-benzoquinonemonoimine), the absorbance maximum of which corresponds to 520 nm.

## 3. Results and Discussion

**Figure 1:**
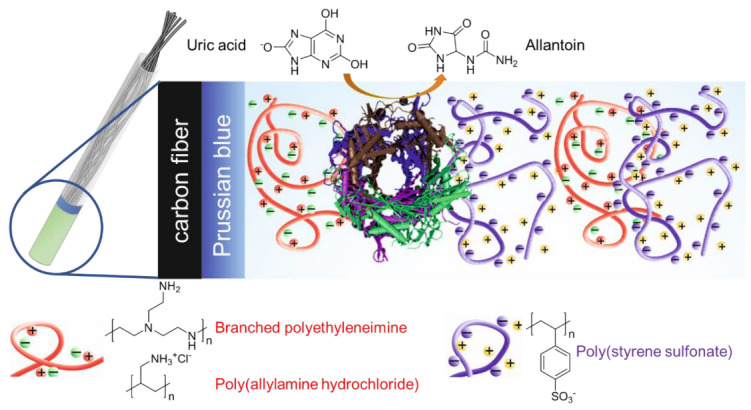
Immobilization of UOx onto CFE with PB redox mediator coated by PEI(PAH)/PSS layers.

The concept of fabrication of the uric acid-selective electrode is given in Figure 1. Along with graphene [30] and fabric substrates [31,32], carbon fiber (CF) is a promising substrate for the development of wearable sensors [15,33,34,35,36,37], with a high strength-to-volume ratio, and was suggested in this work as an electrode for a three-electrode electrochemical cell due to its high carrier mobility, electrical conductivity, environmental stability, low weight, and high-temperature resistance, as well as low-cost production. To perform electrochemical study of enzyme activity, the CF surface needs to be modified through electrodeposition with Prussian blue (Fe_4_[Fe(CN)_6_]_3_) (PB), which is used as a non-diffusion redox mediator and prevents side reactions of metal salts with polyelectrolytes [38,39]. Subsequent modification of the PB-coated CF electrode is aimed at the development of a crowded environment via the hydrogel nanolayer with immobilized UOx, which is a polyelectrolyte complex obtained through LbL assembly of coacervate layers. Weak cationic polyelectrolytes PEI and PAH are usually employed as the first layer [40] and they normally do not form polyelectrolyte coacervate complexes due to a significant relaxation time and interaction with an oppositely charged polyelectrolyte [41]. However, weak polyelectrolytes could adsorb both on positively and negatively charged surfaces due to their amphiphilicity and, thus, stabilize the next layer of UOx that is negatively charged at pH 7.4 [39]. Coating of the uricase layer with the negatively charged strong PSS polyelectrolyte could further stabilize the enzyme and provide a charge-balanced polyelectrolyte–protein–polyelectrolyte complex [42,43] due to its deposition after PEI. This strategy of enzyme immobilization allows biomolecules to preserve their activity on the electrode surface due to charge immobilization instead of covalent grafting [44], which creates a pseudo-homogeneous system on the electrode surface and increases enzyme mobility to interact with the substrate. We suppose that polyelectrolyte complexation increases the sensitivity of analysis. Moreover, PEI–PSS and PAH–PSS complexes facilitate enzyme–substrate interaction and prevent the desorption of protein from the electrode surface, which otherwise damps analytical signal over time [45,46].

**Figure 2:**
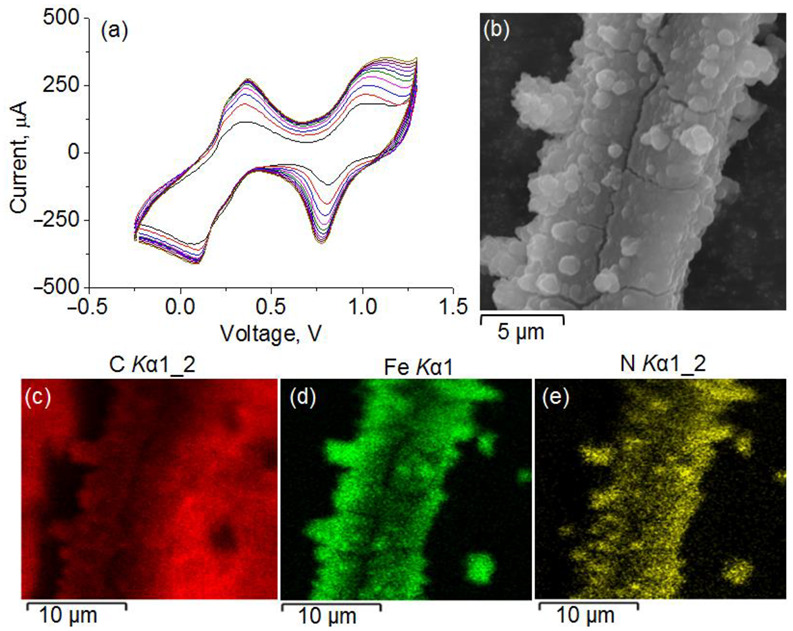
(**a**) Cyclic voltammograms of PB deposition. (**b**) SEM images of the PB-modified CF electrode and corresponding EDX distribution maps of C, Fe, and N atoms.

The surface of the CF electrode was probed by scanning electron microscopy (SEM) and energy-dispersive X-ray spectroscopy (EDX) to evaluate the effectiveness of deposition of PB. SEM images of the CF electrode and uniform distribution of Fe, C, and N in mapped EDX spectra after electrodeposition of PB confirm that the CF surface is fully covered by PB (Figure 2). Voltammogram traces of PB mediator show intrinsic redox peaks centered at ca. +380 mV, which correspond to the oxidation of the ferrocyanide ion.

**Figure 3:**
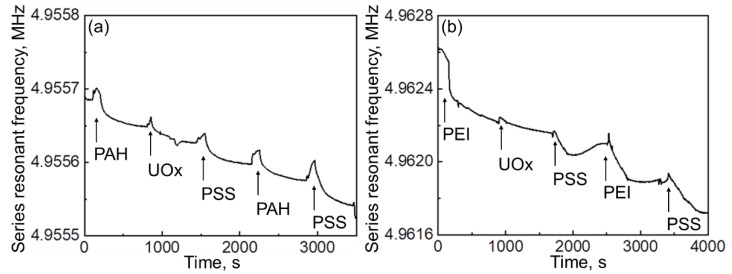
QCM data for adsorbed (**a**) PAH/UOx/PSS/PAH/PSS and (**b**) PEI/UOx/PSS/PEI/PSS layers.

The QCM technique was further employed to evaluate the stability of LbL-assembled enzyme–polyelectrolyte hydrogels at each deposition stage. The amino group in the branched PEI macromolecule is supposed to display a higher adhesion to gold-coated quartz substrate as compared to that in PAH due to less notable steric hindrances. Indeed, deposition of the first layer of branched PEI onto quartz from a flow of corresponding solution in PBS resulted in a mass gain of 0.3839 μg, which is three times as large as that during deposition of the first layer of PAH (0.1468 μg) (Figure 3, Table 1). Subsequent adsorption of UOx and PE layers always led to a weight gain and final washing of the film with PBS did not alter the resonant frequency of quartz, which confirmed the construction of a stable crowded interface through polyelectrolyte layer interactions [47]. Comparison of added mass values after deposition of the second layer of cationic polyelectrolyte onto PSS indicates a stronger interaction between PSS and PEI (+0.5686 μg) as compared to that between PAH and PEI (+0.0645 μg). Sharper peaks on the QCM curve corresponding to the adsorption of PAH/UOx/PSS/PAH/PSS multilayer as compared to broader ones in the case of PEI/UOx/PSS/PEI/PSS suggest the formation of more rigid film on the Au-coated quartz surface when using PAH polyelectrolytes instead of PEI.

**Figure 4:**
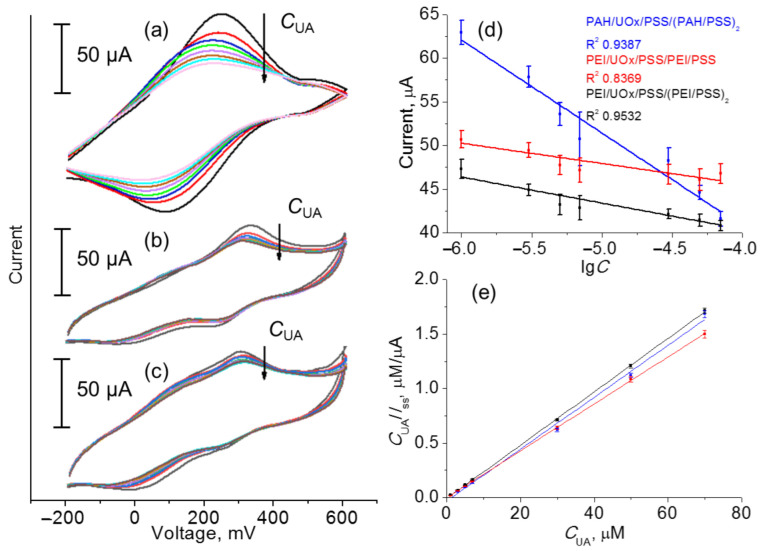
Cyclic voltammogram traces recorded using (**a**) PAH/UOx/PSS/(PAH/PSS)_2_, (**b**) PEI/UOx/PSS/(PEI/PSS)_2_, and (**c**) PEI/UOx/PSS/PEI/PSS working electrodes and (**d**) corresponding calibration plots and (**e**) Hanes–Woolf plots for the calculation of the Michaelis–Menten constant (*K*_m_).

Evidence of the temporally stable LbL-assembled hydrogel made the fabrication of the CF-based working electrode possible and PB/CFE was sequentially coated with a hydrogel nanolayer of PAH–enzyme or PEI–enzyme composition. An interface based on CF, Ag paste, PE layers, and a membrane represented a pseudo-liquid reference electrode, which ensures stable and reproducible analytical signal [15,36]. The electrochemical sensor made from three PE–UOx nanoarchitectures of working electrode (PAH/UOx/PSS/(PAH/PSS)_2_, PEI/UOx/PSS/(PEI/PSS)_2_, and PEI/UOx/PEI/PSS) showed a sufficiently high differentiation between oxidation and reduction peaks in cyclic voltammogram upon addition of uric acid (Figure 4a–c), which indicates reversibility of the redox process. Further calibration of the fabricated electrodes was made by plotting of decreasing oxidation peak current intensity against the increasing concentrations of UA in order to evaluate their relationship (Figure 4a–c). This resulted in a linear response of current in a wide dynamic range of concentration of UA (1–70 μM; Figure 4d). The largest change of the analytical signal represented by the current intensity on the surface of CFE centered at ca. 300 mV, which corresponds to the UA → UOx → [Fe(CN)_6_]^3−^ cascade electron transfer, was reported in the case of the PAH/UOx/PSS/(PAH/PSS)_2_ layer. The slope of the fitted line of current vs. lg*C* plot (10.64 ± 0.96 μA μM^−1^) indicated the highest sensitivity of this electrode configuration (Figure 4d, blue line). It should be noted that the current response of uricase-free polyelectrolyte-modified PB-CFEs to high concentrations of uric acid (70 μM) was exactly the same as that in PBS solution at pH 7.4 (Figure S1), which indicates the role of UOx as a mediator of electron transfer from UA to ferrocyanide ion. The detection limit of all fabricated sensors was further measured at the uric acid concentration, at which signal-to-noise ratio is 3σ, and was estimated to be 0.81 ± 0.07 μM. The values of Michaelis–Menten constant (*K*_m_) of ca. 2 μM were determined from Hanes–Woolf plots (Figure 4e) and confirmed high affinity of the immobilized enzyme to UA, which allows one to use the developed sensors for electrochemical detection of UA. Comparison with the free uricase (340 μM [29]) indicates a higher enzyme activity (by more than two orders of magnitude).

**Figure 5:**
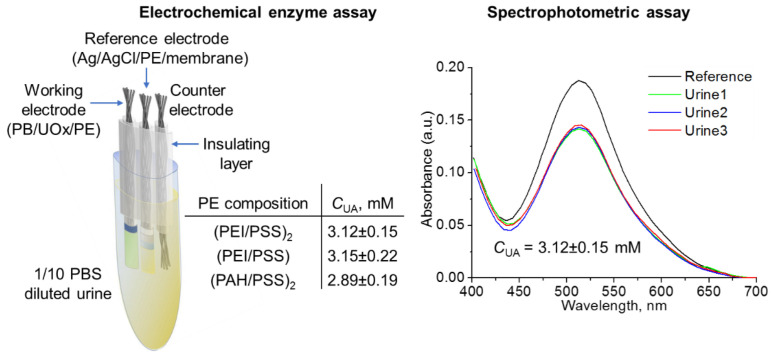
The results of the analysis of UA content using the proposed CF-based three-electrode system and reference values of spectrophotometric assay of urine samples of SARS-CoV-2 patients.

UA detection within hydrogel nanoarchitectures was also probed for urine samples of SARS-CoV-2 patients (Figure 5). The results of analysis using the proposed CF-based three-electrode system and the crowded nanoarchitecture of PE–enzyme composition correlate with the reference values of spectrophotometric assay showing a low variation due to repeatability (standard deviation is 4.8% in the case of PEI/UOx/PSS/(PEI/PSS)_2_ to 7.0% in the case of PAH/UOx/PSS/(PAH/PSS)_2_).

The obtained linear range, sensitivity, limit of detection, and Michaelis–Menten constant values of the fabricated sensor show its competitiveness with analogous enzymatic uric acid electrochemical sensors based on screen-printed electrodes or metal substrates [29,48,49,50,51,52,53,54,55], the main characteristics of which are listed in Table 2, as well as non-enzymatic sensors [56]. Unfortunately, sensor performance data for the electrodes modified with polyelectrolyte multilayers with alternating uricase layers [54] were insufficient for a direct comparison with the sensor in this work, but our fabricated sensor benefits from a lower consumption of uricase enzyme (one layer in this work vs. ten layers in [54]) and a less expensive substrate (CFE vs. Pt). Thus, the CF-based LbL-assembled uricase biosensor is prospective for clinical use due to its low cost, portability, and a relatively simple design.

## 4. Conclusions

An approach to the design of a uric acid sensor based on a carbon fiber electrode coated by uricase enzyme in a crowded medium of synthetic polyelectrolytes through electrostatic interactions has been proposed. The competitiveness of electrochemical enzyme assay with spectrophotometric assay for the determination of uric acid in terms of repeatability has been demonstrated. The proposed carbon fiber system modified with the hydrogel nanolayer of polyelectrolytes can be used to collect big data and apply machine learning for fundamental physicochemical study of proteins in crowded systems and practical application as single-use electrochemical sensors.

## Figures and Tables

**Table 1:** Average mass gain values from two independent QCM measurements.

Polyelectrolyte System	Layer Composition	Added Mass, μg	Final Mass, μg
PAH/UOx/PSS/PAH/PSS	PAH	0.1468	0.1468
	PAH/UOx	0.0544	0.2012
	PAH/UOx/PSS	0.0739	0.2751
	PAH/UOx/PSS/PAH	0.0645	0.3396
	PAH/UOx/PSS/PAH/PSS	0.0890	0.4286
PEI/UOx/PSS/PEI/PSS	PEI	0.3839	0.3839
	PEI/UOx	0.1637	0.5476
	PEI/UOx/PSS	0.1547	0.7023
	PEI/UOx/PSS/PEI	0.5686	1.2709
	PEI/UOx/PSS/PEI/PSS	0.4201	1.6910

**Table 2:** Comparison of analytical characteristics of enzymatic electrochemical sensors of uric acid.

Sensor	Linear Range, μM	LOD, μM	*K*_M_, μM	Sensitivity, μA/μM
UOx/ZnO/ITO [48]	5–750	130	n/a	0.010
Nafion/UOx/ZnO/FTO [49]	10–1500	2.5	n/a	0.345
PANI–UOx/ITO [29]	10–600	10	5.1	0.016–0.047
UOx/PANI–PB/Pt [50]	10–160	2.6	34.13	0.160
UOx/MWCNT/SPE [51]	0.0001–1	0.0005	0.04	418
UOx/MWCNT-CMC/Au [52]	20–5000	2.8	630	0.233
GA/UOx/Chitosan/SACNT/Pt [53]	100–1000	1.0	n/a	0.518
(UOx/PAA)_9_UOx/(PAA/PVS)_2_PAA/Pt [54]	1–1000	n/a	n/a	n/a
UOx/Fc/Cu_2_O/GCE [55]	0.1–1000	0.06	34.74	0.002
This work	1–70	0.81 ± 0.07	2	10.64 ± 0.96

## Data Availability

Not applicable.

## Supplementary Materials

The following supporting information can be downloaded at: https://www.mdpi.com/article/10.3390/polym14235145/s1, Figure S1: Cyclic voltammograms recorded using (PEI/PSS)_2_/PB/CF working electrode in PBS solution or 0.07 mM solution of UA in PBS.

## References

[1] Tan C., Saurabh S., Bruchez M.P., Schwartz R., LeDuc P. (2013). Molecular crowding shapes gene expression in synthetic cellular nanosystems. Nat. Nanotechnol..

[2] Cheung M.S., Klimov D., Thirumalai D. (2005). Molecular crowding enhances native state stability and refolding rates of globular proteins. Proc. Natl. Acad. Sci. USA.

[3] Akabayov B., Akabayov S.R., Lee S.-J., Wagner G., Richardson C.C. (2013). Impact of molecular crowding on DNA replication. Nat. Commun..

[4] Weilandt D.R., Hatzimanikatis V. (2019). Particle-based simulation reveals macromolecular crowding effects on the Michaelis-Menten mechanism. Biophys. J..

[5] Pavlovic M., Plucinski A., Zhang J., Antonietti M., Zeininger L., Schmidt B.V.K.J. (2020). Cascade kinetics in an enzyme-loaded aqueous two-phase system. Langmuir.

[6] Wang Y., Pan T., Wei X., Su F., Li A., Tai Y., Wei T., Zhang Q., Kong D., Zhang C. (2022). Bioinspired enzymatic compartments constructed by spatiotemporally confined in situ self-assembly of catalytic peptide. Commun. Chem..

[7] Rodrigues R.T., Siqueira J.R., Caseli L. (2021). Structural and viscoelastic properties of floating monolayers of a pectinolytic enzyme and their influence on the catalytic properties. J. Colloid Interface Sci..

[8] Muravev A.A., Solovieva S.E., Kochetkov E.N., Mel’nikova N.B., Safiullin R.A., Kadirov M.K., Latypov S.K., Antipin I.S., Konovalov A.I. (2013). Thiacalix[4]monocrowns substituted by sulfur-containing anchoring groups: New ligands for gold surface modification. Macroheterocycles.

[9] Van der Meeren L., Verduijn J., Li J., Verwee E., Krysko D.V., Parakhonskiy B.V., Skirtach A.G. (2021). Encapsulation of cells in gold nanoparticle functionalized hybrid Layer-by-Layer (LbL) hybrid shells—Remote effect of laser light. Appl. Surf. Sci..

[10] Skorb E.V., Andreeva D.V. (2013). Layer-by-layer approaches for formation of smart self-healing materials. Polym. Chem..

[11] Kienle D.F., Chaparro Sosa A.F., Kaar J.L., Schwartz D.K. (2020). Polyelectrolyte multilayers enhance the dry storage and pH stability of physically entrapped enzymes. ACS Appl. Mater. Interfaces.

[12] Abalymov A., Parakhonskiy B., Skirtach A.G. (2020). Polymer- and hybrid-based biomaterials for interstitial, connective, vascular, nerve, visceral and musculoskeletal tissue engineering. Polymers.

[13] Prokopovic V.Z., Vikulina A.S., Sustr D., Shchukina E.M., Shchukin D.G., Volodkin D.V. (2017). Binding mechanism of the model charged dye carboxyfluorescein to hyaluronan/polylysine multilayers. ACS Appl. Mater. Interfaces.

[14] Lai X., Gao G., Watanabe J., Liu H., Shen H. (2017). Hydrophilic polyelectrolyte multilayers improve the ELISA system: Antibody enrichment and blocking free. Polymers.

[15] Stekolshchikova A.A., Radaev A.V., Orlova O.Y., Nikolaev K.G., Skorb E.V. (2019). Thin and flexible ion sensors based on polyelectrolyte multilayers assembled onto the carbon adhesive tape. ACS Omega.

[16] Kumari S., Tiyyagura H.R., Pottathara Y.B., Sadasivuni K.K., Ponnamma D., Douglas T.E.L., Skirtach A.G., Mohan M.K. (2021). Surface functionalization of chitosan as a coating material for orthopaedic applications: A comprehensive review. Carbohydr. Polym..

[17] Qian B., Zheng Z., Michailids M., Fleck N., Bilton M., Song Y., Li G., Shchukin D. (2019). Mussel-inspired self-healing coatings based on polydopamine-coated nanocontainers for corrosion protection. ACS Appl. Mater. Interfaces.

[18] Johnston A.R., Minckler E.D., Shockley M.C.J., Matsushima L.N., Perry S.L., Ayzner A.L. (2022). Conjugated polyelectrolyte-based complex fluids as aqueous exciton transport networks. Angew. Chem. Int. Ed..

[19] Park S., Barnes R., Lin Y., Jeon B.-J., Najafi S., Delaney K.T., Fredrickson G.H., Shea J.-E., Hwang D.S., Han S. (2020). Dehydration entropy drives liquid-liquid phase separation by molecular crowding. Commun. Chem..

[20] Wilcox X.E., Ariola A., Jackson J.R., Slade K.M. (2020). Overlap concentration and the effect of macromolecular crowding on citrate synthase activity. Biochemistry.

[21] Hata Y., Kojima T., Koizumi T., Okura H., Sakai T., Sawada T., Serizawa T. (2017). Enzymatic synthesis of cellulose oligomer hydrogels composed of crystalline nanoribbon networks under macromolecular crowding conditions. ACS Macro Lett..

[22] Ivanov A.S., Nikolaev K.G., Stekolshchikova A.A., Tesfatsion W.T., Yurchenko S.O., Novoselov K.S., Andreeva D.V., Rubtsova M.Y., Vorovitch M.F., Ishmukhametov A.A. (2020). Tick-borne *Encephalitis* electrochemical detection by multilayer perceptron on liquid–metal interface. ACS Appl. Bio Mater..

[23] Baldina A.A., Nikolaev K.G., Ivanov A.S., Nikitina A.A., Rubtsova M.Y., Vorovitch M.F., Ishmukhametov A.A., Egorov A.M., Skorb E.V. (2022). Immunochemical biosensor for single virus particle detection based on molecular crowding polyelectrolyte system. J. Appl. Polym. Sci..

[24] Lavrentev F.V., Rumyantsev I.S., Ivanov A.S., Shilovskikh V.V., Orlova O.Y., Nikolaev K.G., Andreeva D.V., Skorb E.V. (2022). Soft hydrogel actuator for fast machine-learning-assisted bacteria detection. ACS Appl. Mater. Interfaces.

[25] Jia D., Muthukumar M. (2018). Topologically frustrated dynamics of crowded charged macromolecules in charged hydrogels. Nat. Commun..

[26] Testa A., Dindo M., Rebane A.A., Nasouri B., Style R.W., Golestanian R., Dufresne E.R., Laurino P. (2021). Sustained enzymatic activity and flow in crowded protein droplets. Nat. Commun..

[27] Ferreira C., Pinto M.F., Macedo-Ribeiro S., Pereira P.J.B., Rocha F.A., Martins P.M. (2020). Protein crystals as a key for deciphering macromolecular crowding effects on biological reactions. Phys. Chem. Chem. Phys..

[28] Li G., Wu X., Zhou C.-l., Wang Y.-m., Song B., Cheng X.-b., Dong Q.-f., Wang L.-l., You S.-s., Ba Y.-m. (2021). Uric acid as a prognostic factor and critical marker of COVID-19. Sci. Rep..

[29] Arora K., Sumana G., Saxena V., Gupta R.K., Gupta S.K., Yakhmi J.V., Pandey M.K., Chand S., Malhotra B.D. (2007). Improved performance of polyaniline-uricase biosensor. Anal. Chim. Acta.

[30] Singh A., Ahmed A., Sharma A., Arya S. (2022). Graphene and its derivatives: Synthesis and application in the electrochemical detection of analytes in sweat. Biosensors.

[31] Singh A., Sharma A., Arya S. (2022). Human sweat-based wearable glucose sensor on cotton fabric for real-time monitoring. J. Anal. Sci. Technol..

[32] Singh A., Sharma A., Ahmed A., Arya S. (2022). Highly selective and efficient electrochemical sensing of ascorbic acid via CuO/rGO nanocomposites deposited on conductive fabric. Appl. Phys. A.

[33] Lim G.N., Ross A.E. (2019). Purine functional group type and placement modulate the interaction with carbon-fiber microelectrodes. ACS Sens..

[34] Farajikhah S., Innis P.C., Paull B., Wallace G.G., Harris A.R. (2019). Facile development of a fiber-based electrode for highly selective and sensitive detection of dopamine. ACS Sens..

[35] Sweilam M.N., Varcoe J.R., Crean C. (2018). Fabrication and optimization of fiber-based lithium sensor: A step toward wearable sensors for lithium drug monitoring in interstitial fluid. ACS Sens..

[36] Li W., Chen R., Qi W., Cai L., Sun Y., Sun M., Li C., Yang X., Xiang L., Xie D. (2019). Reduced graphene oxide/mesoporous ZnO NSs hybrid fibers for flexible, stretchable, twisted, and wearable NO_2_ e-textile gas sensor. ACS Sens..

[37] Nikolaev K.G., Kalmykov E.V., Shavronskaya D.O., Nikitina A.A., Stekolshchikova A.A., Kosareva E.A., Zenkin A.A., Pantiukhin I.S., Orlova O.Y., Skalny A.V. (2020). ElectroSens platform with a polyelectrolyte-based carbon fiber sensor for point-of-care analysis of Zn in blood and urine. ACS Omega.

[38] Salazar P., Martín M., O’Neill R.D., González-Mora J. (2012). Biosensors based on Prussian Blue modified carbon fibers electrodes for monitoring lactate in the extracellular space of brain tissue. Int. J. Electrochem. Sci..

[39] Piermarini S., Migliorelli D., Volpe G., Massoud R., Pierantozzi A., Cortese C., Palleschi G. (2013). Uricase biosensor based on a screen-printed electrode modified with Prussian blue for detection of uric acid in human blood serum. Sens. Actuators B Chem..

[40] Skorb E.V., Shchukin D.G., Möhwald H., Sviridov D.V. (2009). Photocatalytically-active and photocontrollable coatings based on titania-loaded hybrid sol–gel films. J. Mater. Chem..

[41] Bohmer M.R., Evers O.A., Scheutjens J.M.H.M. (1990). Weak polyelectrolytes between two surfaces: Adsorption and stabilization. Macromolecules.

[42] van der Straeten A., Bratek-Skicki A., Jonas A.M., Fustin C.-A., Dupont-Gillain C. (2018). Integrating proteins in layer-by-layer assemblies independently of their electrical charge. ACS Nano.

[43] Zhang J., Zhu Y., Song J., Yang J., Pan C., Xu T., Zhang L. (2018). Novel balanced charged alginate/PEI polyelectrolyte hydrogel that resists foreign-body reaction. ACS Appl. Mater. Interfaces.

[44] Achazi K., Haag R., Ballauff M., Dernedde J., Kizhakkedathu J.N., Maysinger D., Multhaup D. (2021). Understanding the interaction of polyelectrolyte architectures with proteins and biosystems. Angew. Chem. Int. Ed..

[45] Mauser T., Déjugnat C., Möhwald H., Sukhorukov G.B. (2006). Microcapsules made of weak polyelectrolytes:  templating and stimuli-responsive properties. Langmuir.

[46] Delcea M., Möhwald H., Skirtach A.G. (2011). Stimuli-responsive LbL capsules and nanoshells for drug delivery. Adv. Drug Deliv. Rev..

[47] Cheng C.I., Chang Y.-P., Chu Y.-H. (2012). Biomolecular interactions and tools for their recognition: Focus on the quartz crystal microbalance and its diverse surface chemistries and applications. Chem. Soc. Rev..

[48] Dutta P., Sharma V., Bhardwaj H., Agrawal V.V., Sumana R., Sumana G. (2022). Fabrication of electrochemical biosensor using zinc oxide nanoflowers for the detection of uric acid. MAPAN.

[49] Nagal V., Kumar V., Khan M., AlOmar S.Y., Tripathy N., Singh K., Khosla A., Ahmad N., Hafiz A.K., Ahmad R. (2021). A highly sensitive uric acid biosensor based on vertically arranged ZnO nanorods on a ZnO nanoparticle-seeded electrode. New J. Chem..

[50] Thakur B., Sawant S.N. (2013). Polyaniline/Prussian-Blue-based amperometric biosensor for detection of uric acid. ChemPlusChem.

[51] Cancelliere R., Tinno A.D., Cataldo A., Bellucci S., Micheli L. (2021). Powerful electron-transfer screen-printed platforms as biosensing tools: The case of uric acid biosensor. Biosensors.

[52] Fukuda T., Muguruma H., Iwasa H., Tanaka T., Hiratsuka A., Shimizu T., Tsuji K., Kishimoto T. (2020). Electrochemical determination of uric acid in urine and serum with uricase/carbon nanotube /carboxymethylcellulose electrode. Anal. Biochem..

[53] Yang M., Wang H., Liu P., Cheng J. (2021). A 3D electrochemical biosensor based on Super-Aligned Carbon NanoTube array for point-of-care uric acid monitoring. Biosens. Bioelectron..

[54] Hoshi T., Saiki H., Anzai J.-i. (2003). Amperometric uric acid sensors based on polyelectrolyte multilayer films. Talanta.

[55] Yan Q., Zhi N., Yang L., Xu G., Feng Q., Zhang Q., Sun S. (2020). A highly sensitive uric acid electrochemical biosensor based on a nano-cube cuprous oxide/ferrocene/uricase modified glassy carbon electrode. Sci. Rep..

[56] Gupta J., Arya S., Verma S., Singh A., Sharma A., Singh B., Prerna, Sharma R. (2019). Performance of template-assisted electrodeposited Copper/Cobalt bilayered nanowires as an efficient glucose and Uric acid senor. Mater. Chem. Phys..

