# Supplementary material for: Uricase Crowding via Polyelectrolyte Layers Coacervation for Carbon Fiber-Based Electrochemical Detection of Uric Acid
Source: Polymers (Basel). 2022 Nov 26;14(23):5145. doi: 10.3390/polym14235145 (PMC9739113; doi:10.3390/polym14235145)
Supplement: Supplementary file 1 [file polymers-14-05145-s001.zip › polymers-1984845-supplementary.pdf]

Supporting Information

for

Uricase crowding via polyelectrolyte layers coacervation for carbon fiber-based electrochemical detection of uric acid

By

Anna A. Baldina, Liubov V. Pershina, Ulyana V. Noskova, Anna A. Nikitina, Anton A. Muravev, Ekaterina V. Skorb * and Konstantin G. Nikolaev

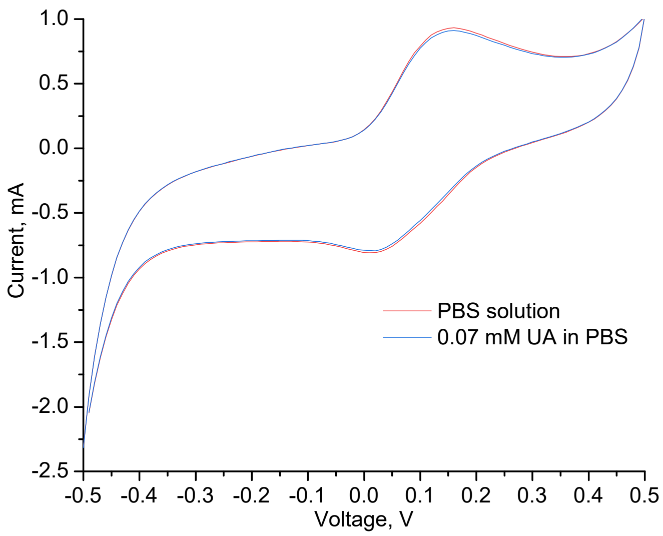

Figure S1. Cyclic voltammograms recorded using $(PEI/PSS)_2$/PB/CF working electrode in PBS solution or 0.07 mM solution of UA in PBS
